# Bibliometric analysis of peer-reviewed literature on Syrian refugees and displaced people (2011–2017)

**DOI:** 10.1186/s13031-018-0179-4

**Published:** 2018-10-29

**Authors:** Waleed M. Sweileh

**Affiliations:** Department of Physiology, Pharmacology/Toxicology, Division of Biomedical Sciences, College of Medicine and Health Sciences, An-Najah National University, Nablus, Palestine

**Keywords:** Syrian refugees, Health, Bibliometric analysis, Research activity

## Abstract

**Background:**

The Syrian conflict has created the worst humanitarian crisis of our time with approximately half of Syria’s pre-war population killed or forced to flee their homes. The current study aimed to analyze peer-reviewed literature published on Syrian refugees and displaced people.

**Methods:**

A bibliometric methodology was implemented using Scopus database after retrieving documents relevant to Syrian refugees and displaced people.

**Findings:**

In total, 323 documents were retrieved. Research articles constituted 71.2% (*n* = 230) of the retrieved documents. The bulk (61.6%; *n* = 199) of the retrieved documents were in health-related fields. Research domains of the retrieved health-related documents were mainly in the field of mental and psychosocial (17.3%; *n* = 56), infectious diseases (15.2%; *n* = 49), health policy and systems (16.4%; *n* = 53), maternal and reproductive health (15.2%; *n* = 49), and non-communicable diseases (NCD) (7.4%; *n* = 24). Authors from research institutions in the United States produced the highest number of publications (24.5%; *n* = 79), followed by Turkey (21.4%; *n* = 69) and Lebanon (10.2%; *n* = 33). The *American University of Beirut* was the most active (5.6%; *n* = 18) research institution. Aside from Lebanon and Jordan, there was very little contribution from other Arab states. *Conflict and Health* was the most active journal (5.3%; *n* = 17) in publishing on Syrian refugees and displaced people.

**Conclusion:**

The study highlighted some particular research gaps – most notably the limited research on NCDs. There was also modest international research collaboration and engagement from Arab countries aside from Lebanon and Jordan.

## Background

There are approximately 68.5 million forcibly displaced people worldwide, the highest number since the Second World War [1]. Among them are nearly 40 million internally displaced, 25.4 million refugees, and 3.1 million asylum-seekers. The increase in the number of forcibly displaced people was mainly caused by the Syrian conflict which was triggered in March 2011 [2]. Currently, there are 6.3 million Syrian refugees and approximately a similar number of internally displaced people. The majority of Syrian refugees live in neighboring countries, particularly Turkey, Lebanon, and Jordan [1].

Research can help inform humanitarian responses to the Syrian crisis, particularly given the high health needs and deliberate targeting of civilians – including use of chemical attacks [3]. While an assessment of research activity on worldwide refugees and internally displaced people has been carried out [4], and also on international Arab migrants in general [5], no study has been carried out specifically to assess literature on Syrian refugees and displaced people to the author’s best knowledge [3]. The aim of this study is to analyze peer-reviewed literature published on Syrian refugees and displaced people.

## Method

The current study applied bibliometric methodology. SciVerse Scopus database was used to achieve the purpose of the study because of the advantages is has over other databases for such studies [6]. In the current study, keywords related to Syrian refugees and displaced people were used to retrieve the relevant documents. The title search methodology, rather than title/abstract, was used to minimize false positive results. The overall search query was as follows: ((((TITLE (syria*) AND TITLE (refugee OR “displaced people” OR “displaced person*” OR refugee OR “asylum seek*” OR exile OR “stateless people” OR “displaced syr*”) AND ALL (cris* OR war OR conflict OR tragedy OR fight* OR violence OR unrest OR uprising OR destruction OR bombing OR *migration OR adlib OR aleppo))) AND NOT TITLE (zaynab OR 2009 OR “Nizip, Turkey” OR “Iraqi refugee*” OR “displaced Iraqi” OR syriac OR “Palestinian refugee*”)) OR ((TITLE-ABS(“syri* refug*”) AND TITLE (syria*)) AND PUBYEAR > 2010 AND PUBYEAR < 2018). The validation of the search strategy was similar to that used in previously published bibliometric studies [5, 7–9].

The retrieved data were exported from Scopus as CSV file and data were analyzed for types of documents, growth of publications, subject areas, most active institution, most active journals, top 10 active countries, number of citations, and research domains related to health. The analysis of research domains was adopted from a previously published bibliometric study on global migration health [8]. All the percentages presented in the current study were calculated from the total number of retrieved documents.

In the current study, international research collaboration was also analyzed based on the presence of different country affiliations for authors of every document. The analysis was performed using specific functions in Scopus and the results were presented as percentage of documents with multiple country affiliation (international collaboration) for each country. The study period was limited from 2011 to 2017. The study was also limited for documents published in peer-reviewed journals. Citation analysis was carried out on June 23rd, 2018.

## Results

In total, 323 documents were retrieved. The types of retrieved documents were research articles (71.2%; *n* = 230), review articles (8.4%; *n* = 27), letters (6.5%; *n* = 21), notes (5.3%; *n* = 17), editorials (4.0%; *n* = 13), conference papers (1.2%; *n* = 4), and undefined type (3.4%; *n* = 11). The retrieved documents were published within different subject areas such as medicine (51.1%; *n* = 165), social sciences (43.7%; 141), arts and humanities (14.2%; *n* = 46), psychology (8.0%; *n* = 26), economics (4.6%; *n* = 15), and microbiology (4.0%; *n* = 13) with potential overlap among the different subject areas. The overall number of documents in health-related field was 199 (61.6%).

Publications started on 2012 with two documents; one was published in *The Lancet* and discussed the living conditions of Syrian refugees during the winter [10] while the second document was published in *Psychologist* and discussed the mental health needs of Syrian refugees [11]. The number of publications grew rapidly after 2014 and reached 115 (35.6%) documents in 2017 (Table 1).

**Table 1:** Number of publications on Syrian refugees and displaced people with time

Year	Number of publications	(%) *N* = 329
2012	2	0.6
2013	21	6.5
2014	22	6.8
2015	61	18.9
2016	102	31.6
2017	115	35.6
Total	323	(100.0)

The retrieved documents received 1181 citations, an average of 3.7 citations per document. The document that received the highest number of citations was published in *International Journal of Psychiatry in Clinical Practice* in 2015 and discussed the post-traumatic stress disorder (PTSD) among Syrian refugees in Turkey [12]. The top 10 cited documents of all retrieved data included three documents in mental and psychosocial health [12–14], one in the field of non-communicable diseases [15], four in the field of infectious diseases [16–19], one in the field of maternal and reproductive health [20], and one in the field of health policy and systems [21].

A deeper analysis indicated that there were 56 (17.3%) documents in mental and psychosocial health, 53 (16.4%) in health policy and systems, 49 (15.2%) in the field of maternal and reproductive health, 49 (15.2%) in the field of infectious diseases, and 24 (7.4%) in the field of non-communicable diseases taking into consideration the presence of certain degree of overlap among the various research domains. It is also important to note that the percentages were calculated from the total number of retrieved documents.

Authors from 39 different countries participated in publishing all retrieved documents. Authors from the USA contributed to 79 (24.5%) documents followed by those from Turkey (21.4%; *n* = 69), and Lebanon (10.2%; *n* = 33) (Table 2). Almost half (50.6%) of the documents authored by the US researchers included co-authors from other countries, particularly from Lebanon. The extent of international research collaboration was as low as 20.3% (*n* = 14) for Turkey and as high as 100% (*n* = 9) for Switzerland. The American University of Beirut (AUB, Lebanon) was the most active institution (5.6%; *n* = 18) followed by the University of Jordan (3.4%; *n* = 11), Johns Hopkins University (2.9%; n = 9), Istanbul University (2.5%; *n* = 8), and Jordan University of Science and Technology (2.5%; n = 8). In total, 906 authors participated in publishing the retrieved documents, a mean of approximately 2.8 authors per document. The retrieved documents included 130 (40.0%) single-authored publications. The retrieved documents were published in 229 different journals. The most active journal in publishing about Syrian refugees was *Conflict and Health* (5.3%; *n* = 17) followed by *The Lancet* (3.7%; *n* = 12) and *Middle East Law and Governance* (3.1%; *n* = 10).

**Table 2:** Top 10 active countries and their international collaboration in the field of Syrian refugees and displaced people

Country	Number of publications (%) *N* = 323	Number of documents with authors having different country affiliation^a^
United States	79 (24.5)	40 (50.6)
Turkey	69 (21.4)	14 (20.3)
Lebanon	33 (10.2)	16 (48.5)
United Kingdom	31 (9.6)	14 (45.2)
Jordan	30 (9.3)	12 (40.0)
Canada	25 (7.7)	10 (40.0)
Germany	16 (5.0)	7 (43.7)
France	9 (2.8)	4 (44.4)
Switzerland	9 (2.8)	9 (100)
Italy	8 (2.5)	4 (50.0)

^a^This represents the international research collaboration for each country. The % was calculated by dividing the numbers in the third column over the numbers in the second column

## Discussion

Research on Syrian refugees and displaced people was dominated by mental and psychosocial health. Health policymakers in host countries need to strengthen the capacity of their health system to face the acute mental health needs of the refugees. Mental health services need to be offered to Syrian refugees at different stages, including after their return and re-settlement. The current study also showed that communicable diseases was another important research domain in the retrieved literature. The devastated health care infrastructure in Syria as well as the overload of healthcare facilities in host countries has hindered immunization programs, drugs, and access to clean water and food supplies. This has led to subsequent emergence of various types of infectious diseases particularly among children [22, 23]. Evidence-based research on prevalence and risk factors of various infections among refugees and internally displaced people should be prioritized. The current study also indicated that the field of NCDs was under-researched given the high burden of NCDs among forcibly displaced Syrians and challenges in accessing care for NCDs. According to UNHCR, 57% of refugees with chronic conditions in Jordan say they cannot afford the care they need for NCDs [24]. There also needs to be more research on health system responses to meet the needs of Syrian refugees and displaced people.

Research in maternal and reproductive health of Syrian displaced people was also relatively low. A study showed that 1 of 3 Syrian women did not seek antenatal care during pregnancy which was reflected in negative birth outcomes [25]. Displaced Syrian women reported lack of access to contraceptive pills which negatively affected the numbers of unwanted pregnancies as well as teen pregnancies and pregnancy complications [26]. Host countries need to increase its healthcare capacities to provide maternal and reproductive health consultation for Syrian refugee women at minimum cost in order to minimize reproductive health problems facing Syrian refugee women.

It should be emphasized here that most of the results and discussion presented in the current study were derived from research publications on Syrian refugees. There is inadequate number of publications on the health of internally displaced Syrians because of the devastated infrastructure in health and education and the difficulty of conducting research under war conditions and limited mobility. Training Syrian healthcare professionals on how to deal with disaster medicine and health consequences of war is of paramount importance. Providing health research support to both Syrians and host countries is key and initiatives such as the Lancet-AUB commission on Syria should be supported in helping to strengthen the science and to mobilize global action [27].

The current study has a few limitations typical of bibliometric methodology [5, 7, 28]. The most important limitation is the fact that several peer-reviewed journals published in Turkey, Jordan, Lebanon and other Middle Eastern countries are not indexed in Scopus and therefore documents published in these journals about Syrian refugees were missed. When interpreting the results of the current study, readers need to be aware that information about authors, institutions, and country affiliation are sometimes missing from the Scopus database or not being updated which creates certain level of errors in subsequent analysis. Finally, despite that the search strategy was validated, the presence of false positive and false negative results remains a possibility.

## Conclusion

The current study highlighted several important points. First, particular health outcomes appear particularly neglected, such as NCDs. Second, there is a need for more research and academic collaboration in the field of Syrian refugees. Third, the involvement of several Arab countries, particularly those in the Arab Gulf, in research about Syrian Refugees was negligible. Finally, there is a need to increase the share of empirical research on Syrian refugees at the expense of editorials, letters, and notes. Research plays an essential role in better understanding the health care needs of displaced Syrians and improving the accessibility, acceptability and effectiveness of health care responses.
